# Experimental Setup for *In Situ* Determination of Conductivity–Porosity–Pressure Relationships during Compression of Solid Electrolytes and of Cathode Active Materials

**DOI:** 10.1021/acsami.6c03789

**Published:** 2026-06-05

**Authors:** Vanessa Miß, Fabio Lange, Stefan Staubitz, Bernhard Roling

**Affiliations:** Department of Chemistry and Marburg Center for Quantum Materials and Sustainable Technologies (mar.quest), 9377 University of Marburg, Hans-Meerwein-Straße 4, Marburg D-35032, Germany

**Keywords:** all-solid-state batteries, solid electrolytes, cathode active materials, *in situ* characterization, pressure-dependent porosity and conductivity

## Abstract

Composite cathodes of all-solid-state batteries (ASSBs) consist of cathode active material (CAM) particles and solid electrolyte (SE) particles. Since ASSBs are typically cycled under external pressure, pressure-dependent electronic transport in the CAM phase and ionic transport in the SE phase play an important role in the battery performance. In order to better understand the relationship between conductivity, porosity, and pressure during the compression of particles, we have built a test station for simultaneous *in situ* measurements of conductivity and porosity under variable pressure. We illustrate the design of this test station, and we show exemplary results for the microcrystalline solid electrolyte Li_5.5_PS_4.5_Cl_1.5_ and for the polycrystalline cathode active material LiNi_0.6_Mn_0.2_Co_0.2_O_2_. The results indicate two distinct porosity regimes: a high-porosity regime, with the conductivity being governed by the interfacial contacts between the particles, and a low-porosity regime with the conductivity being governed by the void space between the particles.

## Graphical abstract

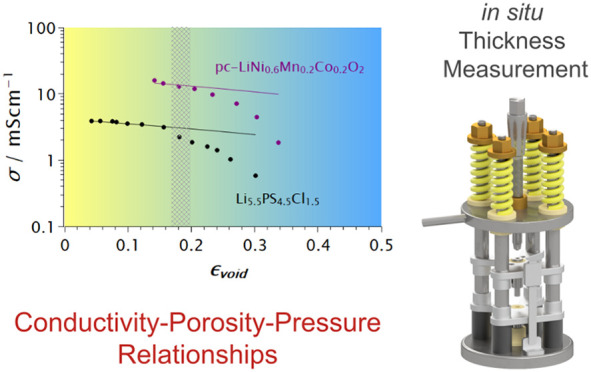

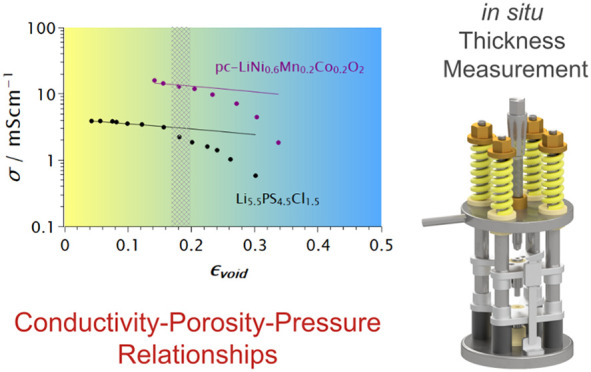

## Introduction

The storage of renewable energies is of great importance in today’s world. Lithium-ion batteries (LIBs) are important storage devices due to their high energy density. However, since the energy density of LIBs is approaching its physicochemical limit, all-solid-state lithium-ion batteries (ASSBs) are considered as a promising alternative. ASSBs can potentially use lithium metal as a negative electrode, leading to an enhanced volumetric energy density.^1^ In addition, ASSBs offer potential advantages in terms of battery safety, since solid electrolytes are nonflammable.^2^

Various classes of solid electrolytes (SEs) have been tested in ASSBs, in particular sulfide-based electrolytes, halide-based electrolytes, oxide-based electrolytes, and polymer electrolytes.^3–8^ Sulfide-based SEs, such as lithium thiophosphates, are considered as particularly promising, as they exhibit high ionic conductivities, are compatible with common anode materials, and can often be synthesized via simple routes.^8–12^ Specifically, high-energy ball milling can be used to synthesize amorphous or glass-ceramic SEs, whereas crystalline SEs can be obtained by employing a heat treatment after ball milling or by means of a solid-state synthesis.^12–16^

In order to assess the potential of a specific SE for the application in ASSBs, precise values for its ionic conductivity are needed. Since the ionic conductivity is strongly dependent on the fabrication pressure of an SE pellet as well as on the stack pressure during the conductivity measurement,^17,18^ a precise control of both pressures is indispensable. Furthermore, precise values for the thickness of the pellet are needed for calculating the ionic conductivity from the measured resistance. In addition, the thickness gives important information about the porosity of the pellet. In literature studies, the porosity of the SE pellets was determined either directly after sample preparation or after finalizing impedance measurements.^19–26^ To this end, two methods were mainly used: (i) determination of the mass and thickness of the pellet in combination with particle density measurement by pycnometry and (ii) FIB/SEM tomography-based reconstruction of the pellet. Thus, in these literature studies, no information was obtained about the influence of the applied stack pressure during the impedance measurements on the porosity and the conductivity of the pellets.

The electronic conductivity of cathode active materials (CAMs), which are mixed with SE particles inside the composite cathode of ASSBs, also plays an important role in the ASSB performance. Common CAMs are transition-metal oxides, such as LiNi_*x*_Mn_*y*_Co_*z*_O_2_ (*x* + *y* + *z* = 1).^27,28^ Since such oxide-based materials are much harder than sulfide-based solid electrolytes, high fabrication pressures are needed to obtain CAM pellets with high electronic conductivity.^29^

In order to obtain accurate values for the ionic conductivity of SEs as well as for the electronic conductivity of CAMs, and in order to analyze conductivity–porosity relationships for SE and CAM pellets, we have developed a new test station for an *in situ* determination of the sample thickness during conductivity measurements under variable pressure. By taking into account the elastic deformation of the pellet, we calculate *in situ* values for the pressure-dependent sample porosities. This novel approach gives insights into conductivity–porosity–pressure relationships *during the compression of pellets*. Herein, we present the design of the test station with pressure control, and we show and discuss exemplary results for the microcrystalline solid electrolyte Li_5.5_PS_4.5_Cl_1.5_ (μc-LPSCl) and for the polycrystalline cathode active material LiNi_0.6_Mn_0.2_Co_0.2_O_2_ (pc-NMC622).

## Experimental Methods

### Sample Preparation

All sample preparations were carried out under an argon atmosphere in a glovebox (UniLab, MBraun, Garching, Germany; *x*_H_2_O_ < 1 ppm and *x*_O_2__ < 1 ppm).

For the synthesis of a 2 g batch of the solid electrolyte Li_5.5_PS_4.5_Cl_1.5_ (μc-LPSCl), stoichiometric amounts of Li_2_S (99.9%, Alfa Aesar, Karlsruhe, Germany), P_2_S_5_ (for synthesis, Sigma-Aldrich, Taufkirchen, Germany), and LiCl (≥99.98%, Sigma-Aldrich, Taufkirchen, Germany) were mixed for 10 min in an agate mortar. Afterward, powder pellets with a diameter of 10 mm were pressed by means of a hydraulic press (P/O/Weber, Remshalden, Germany) with polished stainless steel extrusion dies. A pressure of 196 MPa was applied for 1 min. Then the pellets were transferred into a silica glass ampoule and sealed under vacuum. The ampule was heated in an oven (Nabertherm, Lilienthal, Germany) to 550 °C using a rate of 0.5 °C min^–1^ and was held at 550 °C for 6 h, before it was slowly cooled down. Finally, the pellets were ground in an agate mortar to obtain a crystalline powder.

X-ray diffraction (XRD) measurements on the μc-LPSCl powders were executed using a powder diffractometer STOE STADI MP (STOE, Darmstadt, Germany) and Cu–Kα radiation in a Debye–Scherrer geometry. For this purpose, the powder was filled into a mark tube (Hilgenberg, Malsfeld, Germany) and was closed with a wax under argon.

Commercial polycrystalline LiNi_0.6_Mn_0.2_Co_0.2_O_2_ particles (pc-NMC622, MSE Supplies, Tucson, USA) were used as the CAM.

The powder of the respective material was filled into the measuring cell and closed airtight. Then, the measuring cell was transferred into the test station. The length of the pistons and the thickness of the samples were measured using a micrometer screw with measuring beaks (Mitutoyo, Neuss, Germany). In addition, the length of the springs was measured with a caliper (Holex, Hoffmann Group, Munich, Germany). Two different springs (Febrotec, Halver, Germany; inner diameter 20 mm, outer diameter 40 mm, length 102 mm) were used in the test station. In a pressure range up to 250 MPa, springs with a nominal spring constant of 170.3 N mm^–1^ were chosen, while at higher pressures, springs with a nominal spring constant of 762 N mm^–1^ were chosen. Exact values of the spring constant were obtained by measuring the forces at different spring deflections, see in Figure S1. The impedance spectra of the solid electrolyte and cathode active material were taken at different pressures by means of the NEISYS Potentiostat/Galvanostat/EIS Base Unit (Novocontrol Technologies, Montabaur, Germany) in a frequency range from 1 MHz to 0.1 Hz with an applied AC voltage of 10 mV_rms_. The impedance spectra were analyzed with the software RelaxIS (rhd instruments, Darmstadt, Germany).

Cross-sectional SEM images of pc-NMC622 and μc-LPSCl powder and pellets were taken, which had been prepared at different maximum pressures. The focused ion beam preparation of the cross sections and the SEM imaging were carried out without applied external pressure in a FIB/SEM Crossbeam 550 (Zeiss, Oberkochen, Germany). For the transfer of the powder or the pellets from the glovebox to the FIB/SEM instrument, the powder or the pellets were fixed on a carbon-based sample holder and transferred under inert gas conditions in a sample transfer shuttle (Semilab Conductor Physics Laboratory Co. Ltd., Budapest, Hungary). Cross sections (width: 20 μm, depth: 15 μm) of the pellets were prepared by using a focused gallium ion beam. To this end, a voltage of 30 kV was applied, and the cross-sectional preparation was done in three steps with different ion beam currents (3 nA, 700 pA, 100 pA). The last step had a lower current for a fine polish of the cross section. The SEM images were recorded by applying a voltage of 5 kV and a current of 100 pA using the SE detectors.

### New Test Station and Test Cell

In Figure 1a, a schematic illustration of the measuring cell is shown. The cell consists of two pistons and a polyetheretherketone (PEEK) airtight cell housing, so that measurements can be carried out under an inert gas atmosphere outside a glovebox. Six sealing rings ensure the tightening of the cell, and two lids on each side of the PEEK body prevent the pistons from tilting during the measurements. In addition, the two pistons exhibit a recess for the micrometer screw, which is used for measuring the length of the pistons plus the thickness of the sample, see Figure 1b. We note that a micrometer screw is preferable to a caliper due to the higher precision of the length measurement (5 μm). Furthermore, a groove was milled in the middle of the recess to ensure that the micrometer screw is positioned in a reproducible fashion, see Figure 1c. This facilitates the reproducible handling of the cell considerably. Importantly, the pistons consist of hardened steel in order to avoid any warping of the pistons at high pressures.

**Figure 1:**
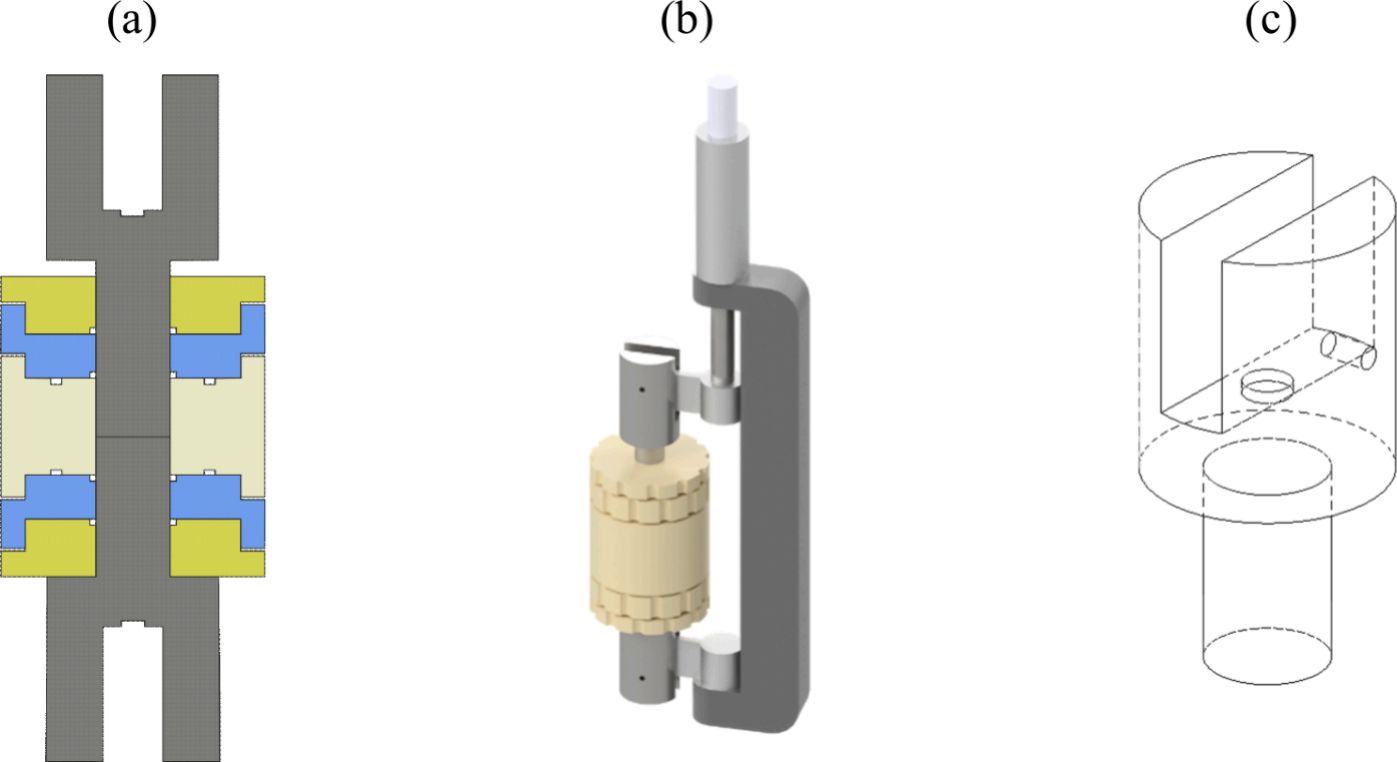
(a) Schematic illustration of the airtight measuring cell; (b) technical drawing of the measuring cell and of the micrometer screw; (c) sketch of the piston with a groove in the middle of the recess ensuring that the micrometer screw is positioned in a reproducible fashion.

In Figure 2a, a schematic illustration of the test station is shown. This station fulfills the following requirements: (i) A guide rail is fitted to prevent the measuring cell from tilting inside the test station. (ii) Four springs are used to ensure the application of a constant pressure to the sample. The spring constants of these springs are in the range of some 100 N mm^–1^, ensuring that changes in pressure due to changes in sample thickness are negligible.

**Figure 2:**
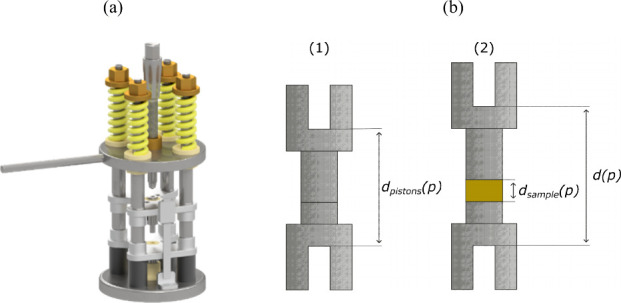
(a) Technical drawing of the test station. (b) Schematic sketch of a sample thickness measurement under an applied pressure *p*.

We note that the *minimum* pressure applicable during the measurements is defined by the mass of the upper piston together with the air pressure and amounts to about *p* = 110 kPa. The spring length at this minimum pressure is determined first. Pressures are then applied using the center screw and the torque wrench, and the change in the spring length is measured.

In order to differentiate between the pressure-dependent thickness of the sample *d*_sample_(*p*) and a reversible piston compression during the measurements, a calibration measurement of the length of the pistons, *d*_pistons_(*p*), under variable pressure is carried out without a sample. During the subsequent measurements on the sample, the sum *d*_pistons_(*p*) + *d*_sample_(*p*) = *d*(*p*) is determined, see Figure 2b, and the pressure-dependent thickness of the sample is calculated via

$$d_{\mathrm{sample}}(p)=d(p)-d_{\mathrm{pistons}}(p)$$ 1

## Results and Discussion

A powder X-ray diffraction pattern shown in Figure S2 verifies the successful synthesis of μc-LPSCl.

In Figure 3, results of measurements on a μc-LPSCl pellet with the following pressure protocol (illustrated in Figure S3a) are shown: A constant pressure was applied to the pellet, and thickness and impedance measurements were carried out. Then, the pressure was released to the minimum pressure of about 110 kPa, and again, thickness and impedance were measured. Subsequently, the applied pressure was increased, measurements were carried out, the pressure was released again to the minimum pressure, measurements were carried out, and so on. After applying the highest pressure of 400 MPa, the pellet was pressed out of the measuring cell, and the thickness of the pressed-out pellet was determined *ex situ* without applied pressure.

**Figure 3:**
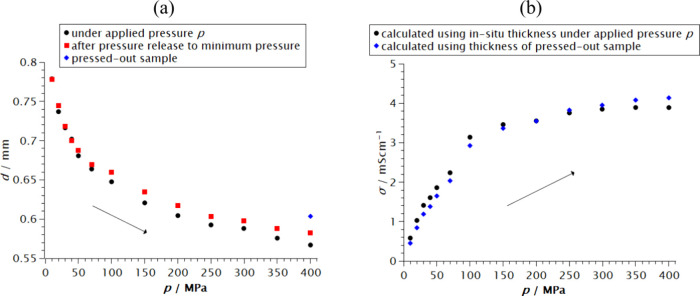
(a) Pressure-dependent thickness of a microcrystalline solid electrolyte Li_5.5_PS_4.5_Cl_1.5_ (μc-LPSCl) pellet at the applied pressure (black), after release to the minimum pressure (110 kPa) in the measuring cell (red), and after pressing the sample out of the measuring cell (blue). (b) Pressure-dependent ionic conductivity of μc-LPSCl calculated using the *in situ* thickness under pressure control (black) and using the thickness of the pressed-out sample (blue).

In Figure 3a, the thickness of a μc-LPSCl pellet calculated by means of eq 1 is plotted versus pressure. The uncertainties of the thickness and pressure data are of the order of the symbol size or lower. The black data points are thicknesses measured at the applied pressure, while the red data points are thicknesses measured after releasing the pressure to the minimum pressure. The blue data point refers to the thickness determined for the pressed-out pellet. The thickness of the sample decreases with increasing applied pressure. After releasing the respective applied pressure to the minimum pressure, the sample thickness increases by 0.3–2.8%. Removal of the pellet from the measuring cell after application of a pressure of 400 MPa leads to a further increase in the sample thickness.

In Figure 3b, results for the pressure-dependent ionic conductivity of a μc-LPSCl pellet are shown. The black dots are conductivity values calculated with the sample thickness determined *in situ* under an applied pressure, while the blue lozenges are values calculated with the thickness of the pressed-out sample. The conductivity values calculated with the *in situ* thickness converge to a constant value of about 4 mS cm^–1^ at pressures above 300 MPa, while the conductivity values calculated with the *ex situ* thickness do not converge. Thus, the *ex situ* thickness value leads to misleading information about the pressure dependence of the ionic conductivity.

In this context, it is important to mention the work by Faka et al. showing that the *material-characteristic* ionic conductivity of argyrodite-type solid electrolytes is characterized by activation volumes between 0.6 and 1.1 cm^3^ mol^–1^.^30^ Such activation volumes lead to a decrease of the material-characteristic ionic conductivity from zero pressure to 400 MPa by 10–20%. In Figure 3b, we show an increase in the measured ionic conductivity by about 1 order of magnitude, and we obtain no indication of a drop in the ionic conductivity with increasing pressure. This gives a strong indication that the pressure dependence of the material-characteristic ionic conductivity is negligible in the pressure range evaluated in this study.

Commercial polycrystalline LiNi_0.6_Mn_0.2_Co_0.2_O_2_ particles (pc-NMC622, MSE Supplies, Tucson, USA) were used as the CAM. Results for the pressure-dependent thickness and electronic conductivity of a pc-NMC622 pellet are shown in Figure S4.

The porosity of the μc-LPSCl pellet and of the pc-NMC622 pellet, ϵ_void_, was determined using the ratio of the density of the pellet under applied pressure ρ­(*p*) and the crystal density of the respective material ρ_0_. ρ­(*p*) was calculated from the mass of pellet *m*, the surface area of the pellet, *A* = π × *r* ^2^, and the *in situ* thickness of the pellet *d*_sample_(*p*) via:

$$\epsilon_{\mathrm{void}}(p)=1-\frac{\rho (p)}{\rho_{0}}=1-\frac{m}{\pi \times r^{2}\times (d_{\mathrm{sample}}(p)+\Delta d_{\mathrm{elastic}}(p))\times \rho_{0}}$$ 2

It is important to note that in eq 2, the elastic deformation of the pellet under the applied uniaxial pressure, Δ*d*_elastic_(*p*) < 0, is taken into account. Δ*d*_elastic_(*p*) is given by

$$\frac{\Delta d_{\mathrm{elastic}}(p)}{d_{\mathrm{sample}}(p=0)}=-\frac{p}{E}$$ 3

with *E* denoting the Young modulus and the pressure *p* corresponding to the uniaxial stress. The elastic deformation Δ*d*_elastic_(*p*) exhibits a negative sign, since the thickness of the pellet decreases under the applied pressure.

Since the minimum pressure is several orders of magnitude lower than the actively applied pressures, *d*_sample_(*p* = 0) can be approximated by the sample thickness measured at the minimum pressure of 0.11 MPa, *d*_0.11 MPa_, leading to

$$\Delta d_{\mathrm{elastic}}=-d_{0.11\;\mathrm{MPa}}\times \frac{p}{E}$$ 4

In Figure 4a, the porosity of both the μc-LPSCl pellet and the pc-NMC622 pellet is plotted versus pressure. For each applied pressure, a data point for ϵ_void_ is shown, obtained at the respective applied pressure (closed symbols) and a second data point obtained after release to the minimum pressure (open symbols). Values for the elastic deformations used for the correction of the sample thickness at the applied pressure are given in Table S1 of the SI. The porosity of μc-LPSCl decreases with increasing applied pressure from about 0.30 at 10 MPa to about 0.04 at 400 MPa. At high pressures, the porosities are much lower than the porosity of a dense packing of spheres, even if these spheres are polydispersive.^31^ This indicates that a strong irreversible deformation of μc-LPSCl particles under high pressures leads to a strong densification of the μc-LPSCl pellet. The porosity of the pc-NMC622 pellet decreases with increasing applied pressure from values of about 0.34 at 50 MPa to about 0.14 at 400 MPa. The ϵ_void_ values obtained at high pressures are also considerably lower than the porosity of a dense packing of polydispersive spheres, suggesting an irreversible deformation of pc-NMC622 particles at high pressures. This is most likely due to a breaking of primary-particle contacts and a rearrangement of primary particles, changing the secondary-particle morphology.

**Figure 4:**
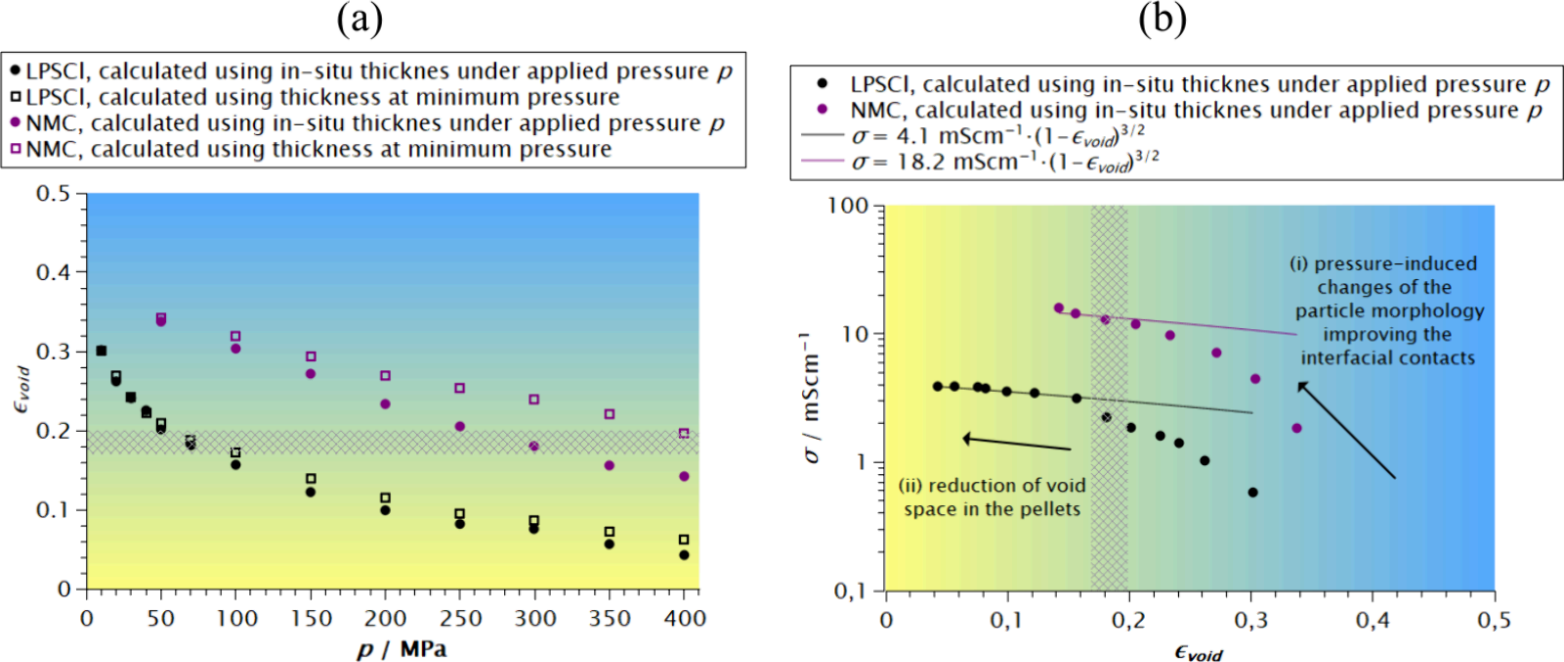
(a) Pressure-dependent porosity ϵ_void_(*p*) of the μc-LPSCl pellet (black) and of the pc-NMC622 pellet (purple). The closed symbols are *in situ* porosities under an applied pressure, while the open symbols are porosities after pressure release to the minimum pressure. (b) Conductivity–porosity relationships for both materials under applied pressure (symbols) and fit with the MWHB theory using eq 5 (lines). In both (a) and (b), the colored background illustrates the two porosity regimes: (i) blue: ϵ_void_ > 0.2; (ii) yellow: ϵ_void_ < 0.17. The shaded area marks the transition between the two regimes.

A comparison of ϵ_void_ calculated under an applied pressure of 400 MPa with values after release to the minimum pressure reveals that the major fraction of the densification of μc-LPSCl is irreversible, while a significant fraction of the densification of pc-NMC622 is reversible.

In Figure 4b, conductivity–porosity relationships are shown for the μc-LPSCl pellet (black data points) and for the pc-NMC622 pellet (violet data points). We have included fits of the data to the predictions of the Maxwell–Wagner–Bruggemann–Hanai (MWBH) theory of composite materials.^32^ In the framework of this effective-medium theory, an insulating phase with volume fraction ϵ_void_, representing the pore space between the particles, is dispersed in a conductive phase with volume fraction 1 – ϵ_void_, representing the μc-LPSCl or the pc-NMC622 particles. The theory predicts the following equation for the conductivity of the respective material in dependence on its porosity ϵ_void_:

$$\sigma (\epsilon_{\mathrm{void}})=\sigma_{0}\times (1-\epsilon_{\mathrm{void}}{)}^{3/2}$$ 5

with σ­(ϵ_void_) and σ_0_ denoting the porosity-dependent conductivity of the pellet and the conductivity of a void-free pellet, respectively. The MWBH theory accounts for the weak increase of σ­(ϵ_void_) with decreasing porosity at porosities ϵ_void_ < 0.2, but fails to reproduce the strong increase of σ­(ϵ_void_) with decreasing porosity at porosities ϵ_void_ > 0.2.

In order to elucidate the origin of these deviations from the MWBH theory at low pressures and high porosities, we have prepared cross sections of the pellets by focused ion beam (FIB) milling and have taken cross-sectional SEM images, see Figure 5. The pellets were fabricated using different fabrication pressures, which, according to Figure 4a, correspond to different porosities ϵ_void_. Without applied pressure, the particles of a pc-NMC622 powder exhibit a morphology that is close to spherical, see Figure 5 (left). After applying a pressure of 50 MPa (corresponding to ϵ_void_ = 0.33), the morphology is still close to spherical, but slight deformations close to particle–particle contacts are already visible. After increasing the pressure to 200 MPa (corresponding to ϵ_void_ = 0.23), significant changes in the morphology of the secondary particles are detectable. Since the SEM images were taken without applied pressure, the void space between the surfaces of neighboring secondary particles is visible. However, the inverse surface topography of neighboring particles and, in particular, the flattening of the secondary NMC622 particle surfaces close to interfaces (see white lines) gives a strong indication for good interfacial contacts between these particles under applied pressure. Such pressure-induced changes of the secondary-particle morphology, improving the interfacial contacts, are not taken into account in the effective-medium MWBH theory and lead to a stronger dependence of the electronic conductivity on the porosity than predicted by the theory. We note that in this porosity regime ϵ_void_ > 0.2 (blue background color in Figure 4), there should also be a reduction of void space between particles, which does not significantly affect the interfacial contacts. However, as compared to the interfacial contact effects, this reduction of void space exerts only a weak influence on the electronic conductivity. Once good interfacial contacts are established, a further increase of the pressure from 200 to 400 MPa (corresponding to ϵ_void_ = 0.14) leads mainly to a reduction of the void space in the pellets. Therefore, it is plausible that the interrelation between porosity and conductivity in this regime (yellow background color in Figure 4) is well described by the MWBH theory. On the left-hand side of Figure 6, we sketch the pressure-induced changes of the interfacial contacts and the pressure-induced reduction of void space in the two distinct regimes of the σ­(ϵ_void_) data.

**Figure 5:**
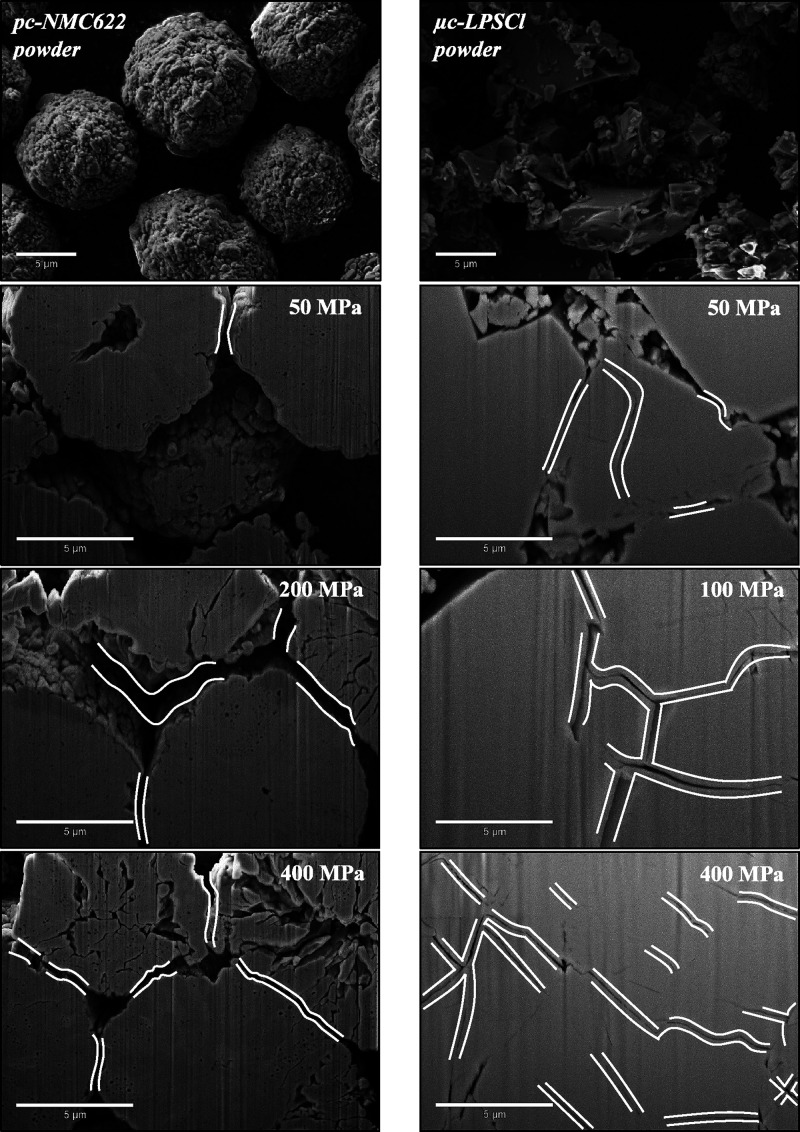
(Left) SEM images of pc-NMC622 powder and cross-sectional SEM images of pc-NMC622 pellets after compression at different pressures. (Right) SEM images of μc-LPSCl powder and cross-sectional SEM images of μc-LPSCl pellets after compression at different pressures. The white lines are guides to the eye for pressure-induced changes of the particle morphology improving the interfacial contacts.

**Figure 6:**
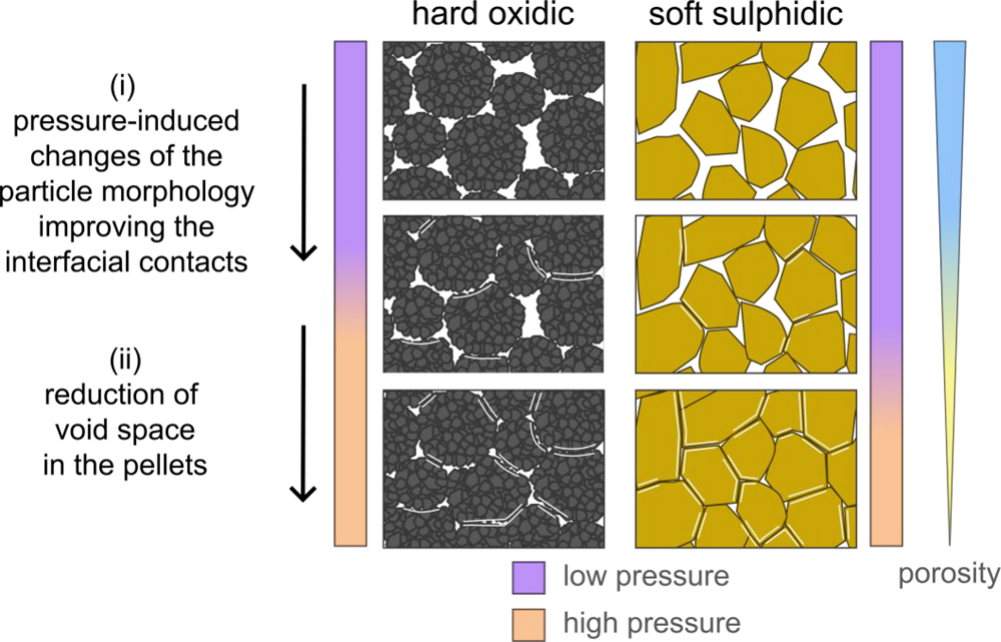
Schematic illustration of pressure-induced changes in the particle morphology and void space of (left) pc-NMC622 and (right) μc-LPSCl.

In the case of μc-LPSCl, the σ­(ϵ_void_) data obtained at porosities ϵ_void_ ≤ 0.17 (yellow background color in Figure 4) are well described by the MWBH theory. However, the data points at porosities ϵ_void_ > 0.17 (blue background color in Figure 4) deviate strongly from the theory, indicating that the relevance of interfacial contact effects at pressures below 100 MPa, see Figure 5 (right) and schematic illustration in Figure 6.

## Conclusions

In conclusion, we have presented a test station for simultaneous *in situ* measurements of conductivity and pellet porosity under variable pressure. Our results indicate that the conductivity–porosity relationship σ­(ϵ_void_) of pc-NMC622 pellets and μc-LPSCl pellets is characterized by two distinct regimes: (i) A high-porosity regime with ϵ_void_ > 0.17–0.2, in which pellet compression leads to a strong increase of the conductivity due to improved interfacial contacts of particles; and (ii) a low-porosity regime with ϵ_void_ < 0.17–0.2, in which pellet compression leads to a weak increase of the conductivity due to a reduction of the void space inside the pellet. Overall, our results show that the *in situ* determination of the porosity of the pellets and the analysis of conductivity/porosity relationships give important insights into the charge carrier transport in SE and CAM pellets. The application of this method to composite cathodes will be the subject of future work.

## Supplementary Material



## Notes

Determination of the spring constant; powder XRD pattern of μc-Li_5.5_PS_4.5_Cl_1.5_; pressure protocol for *in situ* measurements; pressure-dependent thickness and electronic conductivity of the cathode active material pc-NMC622; additional cross-sectional SEM images of pc-NMC622 and μc-Li_5.5_PS_4.5_Cl_1.5_ pellets; characteristic data of pc-NMC622 and μc-LPSCl pellets; and calculation of the elastic deformation of the pellets (PDF)
