# Supplementary material for: Experimental Setup for In Situ Determination of Conductivity–Porosity–Pressure Relationships during Compression of Solid Electrolytes and of Cathode Active Materials
Source: ACS Appl Mater Interfaces. 2026 Jun 5;18(23):32830–6. doi: 10.1021/acsami.6c03789 (PMC13288386; doi:10.1021/acsami.6c03789)
Supplement: Supplementary file 1 [file am6c03789_si_001.pdf]

# Supporting Information

## Experimental Setup for *In Situ* Determination of Conductivity-Porosity-Pressure Relationships during Compression of Solid Electrolytes and of Cathode Active Materials

Vanessa Miß, Fabio Lange, Stefan Staubitz, Bernhard Roling*

*Department of Chemistry and*
*Marburg Center for Quantum Materials and Sustainable Technologies (mar.quest),*
*University of Marburg, Hans-Meerwein-Straße 4, D-35032 Marburg, Germany*
**Correspondence: roling@staff.uni-marburg.de*

## Determination of the spring constant

Two types of springs were used, a softer type with a nominal spring constant of (170 ± 10%) N $mm^{-1}$ and a stiffer type with a nominal spring constant of (762 ± 10%) N $mm^{-1}$. In order to verify the nominal values given by the manufacturer (Febrotec GmbH, Halver, Germany), the springs were positioned in a hydraulic press (P/O/Weber, Remshalden, Germany), and the spring deflections at different forces were measured with a calliper (Holex, Hoffmann Group, Munich, Germany). This was done for all four springs of the respective spring type. Fig. S1 (a) shows results for the softer spring and (b) results for the stiffer spring. The mechanical properties of all springs follow Hook's law. Accordingly, the respective spring constant can be obtained from the slope of the force vs. deflection data. The results show that the spring constants of the softer springs are slightly higher than the mean value given by manufacturer and the spring constants of the stiff springs are slightly softer than the mean value given by the manufacturer. However, all springs constants are within the manufacturer's tolerance range.

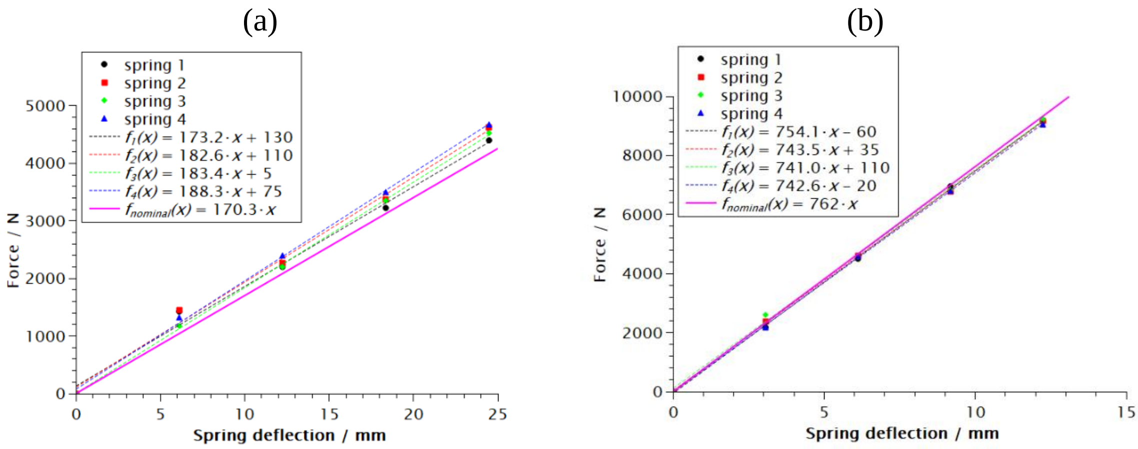


Figure S1. Comparison of the spring characteristic of the soft (a) and stiff (b) springs with the manufacturer specifications $f_{nominal}(x)$

## Results

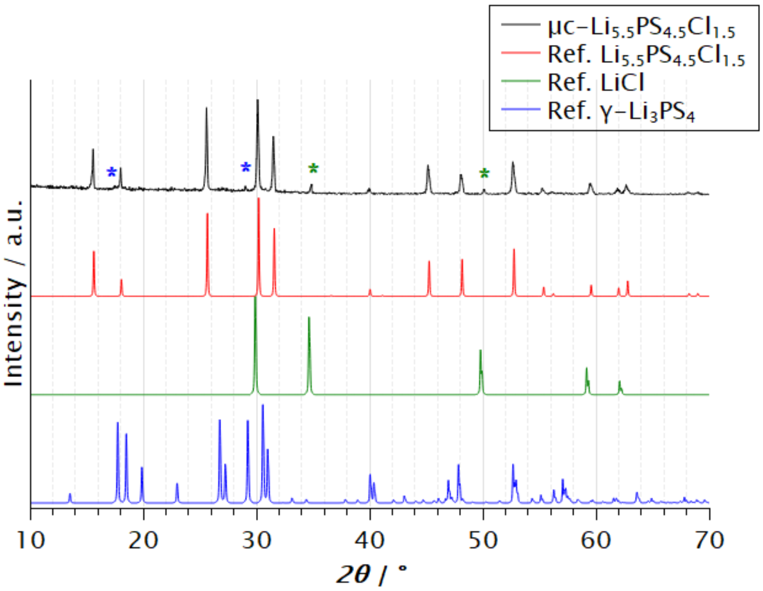


Figure S2. Powder XRD pattern of $\mu$c-$Li_{5.5}PS_{4.5}Cl_{1.5}$ (black) with the references of $Li_{5.5}PS_{4.5}Cl_{1.5}$ (red),[1] LiCl (green)[2] and γ-$Li_3PS_4$ (blue).[3]

(a) (b)

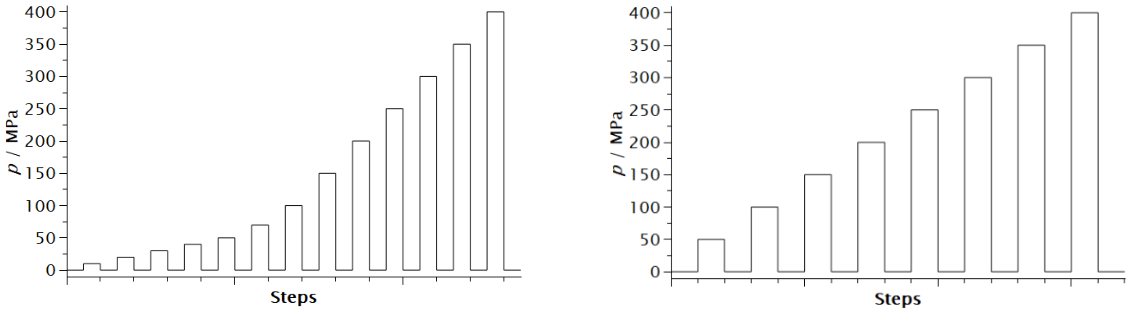


Figure S3. Pressure protocol for the in-situ thickness and impedance measurements of (a) $\mu$c-LPSCl and (b) pc-NMC622 in the new test station. The measurements were carried out during the constant-pressure intervals.

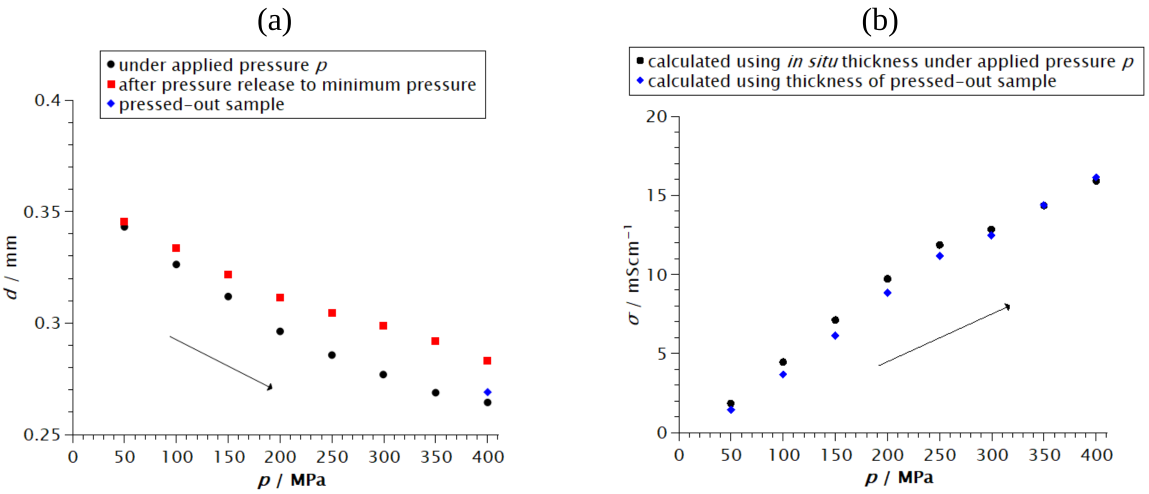


Figure S4. (a) Pressure-dependent thickness of the cathode active material pc-NMC622 under an applied pressure (black) and after pressure release to the minimum pressure of 110 kPa inside the measuring cell (red) as well as after pressing-out the sample of the measuring cell (blue). (b) Pressure-dependent electronic conductivity of the cathode active material pc-NMC622 calculated using the *in situ* thickness under applied pressure (black) and using the thickness of the pressed-out sample (blue). In Miß et al., we have shown that the increase of the electronic conductivity during the compression of a pc-NMC622 pellet is an irreversible process.[4] During pressure release, the electronic conductivity is virtually constant (expect at very low pressures, at which sample/electrode interfacial resistances become relevant). In contrast, the pressure dependence of the material-characteristic electronic conductivity is a reversible phenomenon. Consequently, the virtually constant electronic conductivity during pressure release gives strong indication that the pressure dependence of the material-characteristic electronic conductivity is negligible in the pressure range of our study.

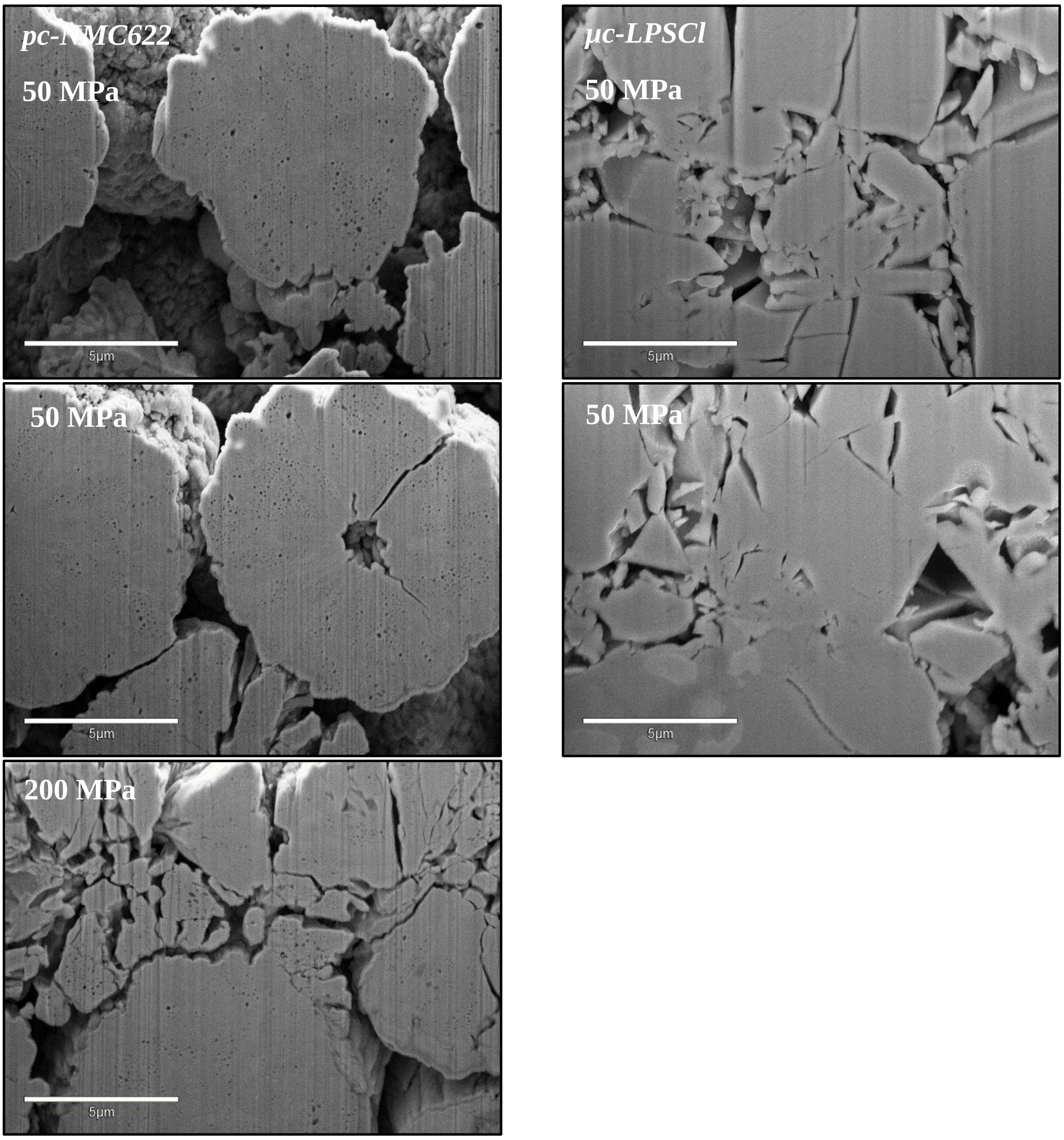


Figure S5. (Left) Additional cross-sectional SEM images of pc-NMC622 pellets after compression at 50 MPa and at 200 MPa, respectively. (Right) Additional cross-sectional SEM images of μc-LPSCl pellets after compression at 50 MPa.

Table S1. Characteristic data of $\mu$c-$Li_{5.5}PS_{4.5}Cl_{1.5}$ and pc-$LiNi_{0.6}Mn_{0.2}Co_{0.2}O_2$ pellets.

| | **$\mu$c-$Li_{5.5}PS_{4.5}Cl_{1.5}$** | **pc-$LiNi_{0.6}Mn_{0.2}Co_{0.2}O_2$** |
|---|---|---|
| Mass of particles in a single pellet<br>$\boldsymbol{m}$ / mg | 80.2 | 80.0 |
| Diameter of the pellet<br>$\boldsymbol{d}$ / cm | 0.5 | 0.5 |
| Mass density of the particles<br>$\boldsymbol{\rho_0}$ / g $cm^{-3}$ | 1.87[1] | 4.48[5] |
| Young's modulus<br>$\boldsymbol{E}$ / GPa | 67[6] | 180[7] |

Table S2. Calculation of the elastic deformation of the pellets, $\Delta d_{strain}$, under different applied pressures $p$.

| **$\boldsymbol{p}$ / MPa** | **$\boldsymbol{d}$ at $x$ MPa / mm** | **$\boldsymbol{d}$ at minimum pressure / mm** | **$\Delta \boldsymbol{d}_{strain}$ / mm** |
|---|---|---|---|
| **$\mu$c-$Li_{5.5}PS_{4.5}Cl_{1.5}$** | | | |
| 10 | 0.77875 | 0.778125 | −0.000116 |
| 20 | 0.736875 | 0.744375 | −0.000222 |
| 30 | 0.71625 | 0.718125 | −0.000322 |
| 40 | 0.701875 | 0.7 | −0.000418 |
| 50 | 0.680625 | 0.6875 | −0.000513 |
| 70 | 0.66375 | 0.669375 | −0-000700 |
| 100 | 0.6475 | 0.66 | −0.000985 |
| 150 | 0.620625 | 0.634375 | −0.001421 |
| 200 | 0.604375 | 0.6175 | −0.001844 |
| 250 | 0.5925 | 0.603125 | −0.002251 |
| 300 | 0.588 | 0.5975 | −0.002671 |
| 350 | 0.575625 | 0.588125 | −0.003072 |
| 400 | 0.566875 | 0.5825 | −0.003478 |
| **pc-$LiNi_{0.6}Mn_{0.2}Co_{0.2}O_2$** | | | |
| 50 | 0.343125 | 0.345625 | −0.000096 |
| 100 | 0.32625 | 0.33375 | −0.000185 |
| 150 | 0.311875 | 0.321875 | −0.000268 |
| 200 | 0.29625 | 0.31125 | −0.000346 |
| 250 | 0.285625 | 0.304375 | −0.000423 |
| 300 | 0.276875 | 0.29875 | −0.000497 |
| 350 | 0.26875 | 0.291875 | −0.000567 |
| 400 | 0.264375 | 0.283125 | −0.000629 |

## References


(1) Adeli, P.; Bazak, J. D.; Park, K. H.; Kochetkov, I.; Huq, A.; Goward, G. R.; Nazar, L. F. Superionic Conductor Boosting Solid-State Diffusivity and Conductivity in Lithium Superionic Argyrodites by Halide Substitution. *Angew. Chemie* **2019**, *58*, 8681–8686.

(2) Posnjak, E.; Wyckoff, R. W. G. The Crystal Structures of the Alkali Halides . II. *J. Washingt. Acad. Sci.* **1922**, *12* (10), 248–251.

(3) Homma, K.; Yonemura, M.; Kobayashi, T.; Nagao, M.; Hirayama, M.; Kanno, R. Crystal Structure and Phase Transitions of the Lithium Ionic Conductor $Li_3PS_4$. *Solid State Ionics* **2011**, *182*, 53–58.

(4) Miß, V.; Seus, S.; Marx, A.; Steyer, E. D.; Mereacre, V.; Binder, J. R.; Roling, B. Influence of Pressure, Particle Morphology, Coating, and Heat Treatment on the Effective Electronic Conductivity of Cathode Active Materials for All-Solid-State Batteries. *ACS Mater. Lett.* **2025**, *7* (6), 2262–2269.

(5) Hua, W.; Wang, K.; Knapp, M.; Schwarz, B.; Wang, S.; Liu, H.; Lai, J.; Müller, M.; Schökel, A.; Missyul, A.; Ferreira Sanchez, D.; Guo, X.; Binder, J. R.; Xiong, J.; Indris, S.; Ehrenberg, H. Chemical and Structural Evolution during the Synthesis of Layered $Li(Ni,Co,Mn)O_2$Oxides. *Chem. Mater.* **2020**, *32* (12), 4984–4997.

(6) Munsif, M.; Shah, M.; Ullah, Z.; Ashraf, M. W.; Fayaz, M.; Alsalmah, H. A.; Murtaza, G. First Principles Study of the Structural, Mechanical and Optical Properties of $Li_6PS_5X$ (X= Cl, I) Argyrodite Compounds. *Phys. B Condens. Matter* **2023**, *661*, 414932.

(7) Liu, J.; Lin, W.; Wang, Z.; Wang, Y.; Chen, T.; Zheng, J. Elastic Mechanics Study of Layered $Li(Ni_xMn_yCo_z)O_2$. *PRX Energy* **2024**, *3*, 013012.
